# Editorial: Novel targets in pediatrics: advances in diagnostic and therapeutic approaches

**DOI:** 10.3389/fped.2026.1803033

**Published:** 2026-02-27

**Authors:** Francesco Catamerò, Francesca Conti, Silvia Ricci, Martina Votto, Francesco Pegoraro

**Affiliations:** 1 Department of Health Sciences, University of Florence, Florence, Italy; 2 Department of Medical and Surgical Sciences (DIMEC), University of Bologna, Bologna, Italy; 3 Pediatric Unit, IRCCS Azienda Ospedaliero-Universitaria di Bologna, Bologna, Italy; 4 Immunology Unit, Meyer Children’s Hospital IRCCS, Florence, Italy; 5 Department of Clinical, Surgical, Diagnostic and Pediatric Sciences, University of Pavia, Pavia, Italy; 6 Department of Experimental and Clinical Medicine, University of Florence, Florence, Italy; 7 Department of Hematology and Oncology, Meyer Children’s Hospital IRCCS, Florence, Italy

**Keywords:** diagnosis, novel aspects, pediatrics, rare diseases, treatments

Childhood is a uniquely dynamic and biologically sensitive phase of life during which many diseases—particularly rare, immune-mediated, infectious, and genetically driven conditions—first manifest. Yet, despite this critical window of vulnerability and opportunity, pediatric medicine has historically evolved in the shadow of adult research. Therapeutic strategies are often introduced into pediatric practice only after adult validation, and diagnostic tools are frequently adapted rather than specifically developed for children. Ethical complexities, limited patient numbers, regulatory barriers, and concerns regarding fragility have all contributed to a persistent evidence gap. Consequently, children are frequently exposed to treatments whose age-specific safety, pharmacodynamics, and long-term developmental implications remain incompletely defined.

Similarly, diagnostic pathways in pediatrics are often constrained by concerns related to invasiveness, radiation exposure, sedation requirements, and limited pediatric validation studies. However, pediatric medicine is now entering a transformative era. The expansion of targeted therapies, orphan drugs, molecular diagnostics, advanced imaging modalities, bioinformatic platforms, and machine-learning algorithms is redefining what is possible in pediatric care. The 18 contributions included in this Research Topic collectively embody this paradigm shift, spanning rare diseases, infectious and inflammatory conditions, neonatology, critical care, imaging, bioinformatics, digital health, and predictive modeling (Gong et al., Schnapperet al., Zheng etal., Cai et al., Ghirigato et al., Iwayama et al., Yi et al., Lee et al., Cao et al., Shen et al., Salerno et al., Wang Y et al., Wang L et al., Sun et al., Yang et al., Xie et al., Zhu et al., Krishnegowda et al.).

## Rare diseases and molecular insights: toward truly personalized pediatrics

Rare diseases remain one of the most compelling frontiers in pediatric medicine. The case report describing infantile fibrosarcoma with a BRAF gene mutation exemplifies the clinical relevance of molecular characterization in rare pediatric tumors. By identifying a non-classical mutation in a gastrointestinal presentation, the study underscores how genomic profiling can refine diagnostic accuracy and inform surgical and oncologic strategies.

Equally illustrative is the report on acquired methemoglobinemia in early infancy, a rare but potentially life-threatening condition requiring rapid biochemical confirmation and targeted therapy. Pediatric-onset relapsing polychondritis with airway involvement further reinforces the importance of individualized management strategies in rare inflammatory diseases, integrating surgical innovation with systemic immunomodulation.

Other rare or complex presentations discussed in this Research Topic include acute suppurative appendicitis complicated by gallbladder torsion, highlighting the importance of intraoperative vigilance, and atypical Campylobacter pseudo-appendicitis mimicking surgical abdomen, emphasizing diagnostic refinement to avoid unnecessary procedures.

## Advancing diagnostic pathways: safer, smarter, and child-specific

Diagnostic innovation is a recurring theme. The study exploring contrast-enhanced ultrasound in pediatric acute kidney injury provides a radiation-free method to evaluate renal microcirculation and correlate perfusion patterns with pathophysiology (Iwayama et al.). Similarly, the randomized controlled trial assessing parental presence during pediatric MRI examinations demonstrates how procedural optimization can reduce sedation requirements and enhance safety.

The systematic review and meta-analysis evaluating the predictive value of the direct antiglobulin test for neonatal hyperbilirubinemia critically reassesses a widely used screening tool, advocating for risk-based application rather than routine testing.

In infectious diseases, a machine learning–based diagnostic model for severe Mycoplasma pneumoniae pneumonia illustrates how laboratory and clinical markers can be integrated to improve early risk stratification.

## Molecular medicine and systems biology in pediatric disease

The application of bioinformatics and transcriptomics is exemplified by the study identifying key genes in pediatric sepsis using weighted gene coexpression network analysis. By distinguishing pediatric-specific molecular signatures from adult datasets, this work reinforces the principle that pediatric immune responses are biologically distinct and require dedicated investigation.

Predictive modeling is further advanced by the development of a machine learning–based nomogram for juvenile idiopathic arthritis screening using large national datasets. This tool aims to support early recognition of chronic inflammatory disease in children.

Similarly, the predictive model for Henoch–Schönlein purpura nephritis identifies demographic and clinical risk factors associated with renal involvement, offering a structured framework for closer monitoring of high-risk patients.

## Therapeutic innovation across the pediatric continuum

Therapeutic advancement is represented across multiple subspecialties. The systematic review and meta-analysis on neurally adjusted ventilatory assist in pediatric intensive care units evaluates advanced ventilation strategies aimed at optimizing respiratory support and reducing complications.

In palliative care, the scoping review on alpha-2 agonists for refractory neurological symptoms explores emerging pharmacologic options for symptom control in children with life-limiting conditions.

Emergency medicine is addressed through the systematic review and meta-analysis on migraine treatment in pediatric emergency departments, which synthesizes evidence to support more standardized and effective acute management.

## Digital health, longitudinal care, and quality of life

Innovation in pediatrics extends beyond acute care to longitudinal follow-up and patient-centered outcomes. The study on intelligent interactive systems for comprehensive care of very-low-birth-weight infants demonstrates how digital platforms can improve neurodevelopmental monitoring, growth outcomes, and parental satisfaction.

Quality of life assessment is critically examined in the systematic review of QoL tools for chronic cough in children, emphasizing the need for validated and culturally adapted instruments in pediatric outcome measurement.

Broader population-based predictive analytics are represented by the development of a machine learning–based nomogram for early screening of juvenile idiopathic arthritis in large U.S. datasets, demonstrating how big data can inform early identification strategies, alongside risk prediction frameworks for inflammatory and nephrological complications.

## A unified vision: toward evidence-based pediatric precision medicine

Collectively, the 18 articles in this Research Topic provide a cohesive vision of pediatric medicine at a critical juncture. From molecular characterization of rare tumors and immune dysregulation in sepsis, to predictive nomograms for chronic inflammatory diseases, advanced imaging, sedation-reduction strategies, digital longitudinal care, and quality-of-life evaluation, the contributions illustrate the convergence of technology, molecular insight, and clinical pragmatism.

They affirm that the future of pediatrics lies not in extrapolating adult frameworks but in establishing independent, developmentally informed, and data-driven strategies tailored to children's unique biology and life course. Through sustained innovation, critical appraisal of existing practices (Lee et al.), and responsible integration of artificial intelligence, pediatric healthcare can progress toward a safer, more precise, and more equitable future for children worldwide.

